# Using Mobile Virtual Reality Simulation to Prepare for In-Person Helping Babies Breathe Training: Secondary Analysis of a Randomized Controlled Trial (the eHBB/mHBS Trial)

**DOI:** 10.2196/37297

**Published:** 2022-09-12

**Authors:** Beatrice Nkolika Ezenwa, Rachel Umoren, Iretiola Bamikeolu Fajolu, Daniel S Hippe, Sherri Bucher, Saptarshi Purkayastha, Felicitas Okwako, Fabian Esamai, John B Feltner, Olubukola Olawuyi, Annet Mmboga, Mary Concepta Nafula, Chris Paton, Veronica Chinyere Ezeaka

**Affiliations:** 1 Department of Paediatrics College of Medicine University of Lagos Lagos Nigeria; 2 Department of Pediatrics University of Washington Washington, WA United States; 3 Clinical Research Division Fred Hutchinson Cancer Center Washington, WA United States; 4 Department of Pediatrics Indiana University School of Medicine Indiana, IN United States; 5 Department of BioHealth Informatics Indiana University-Purdue University at Indianapolis Indianapolis, IN United States; 6 Department of Paediatrics Alupe University College Busia Kenya; 7 Centre for Tropical Medicine Nuffield Department of Clinical Medicine Oxford United Kingdom

**Keywords:** virtual reality, mobile learning, Helping Babies Breathe, neonatal resuscitation, mobile Helping Babies Survive powered by District Health Information Software 2, neonatal mortality, digital education, health care education, health care worker, medical education, digital intervention

## Abstract

**Background:**

Neonatal mortality accounts for approximately 46% of global under-5 child mortality. The widespread access to mobile devices in low- and middle-income countries has enabled innovations, such as mobile virtual reality (VR), to be leveraged in simulation education for health care workers.

**Objective:**

This study explores the feasibility and educational efficacy of using mobile VR for the precourse preparation of health care professionals in neonatal resuscitation training.

**Methods:**

Health care professionals in obstetrics and newborn care units at 20 secondary and tertiary health care facilities in Lagos, Nigeria, and Busia, Western Kenya, who had not received training in Helping Babies Breathe (HBB) within the past 1 year were randomized to access the electronic HBB VR simulation and digitized HBB Provider’s Guide (VR group) or the digitized HBB Provider’s Guide only (control group). A sample size of 91 participants per group was calculated based on the main study protocol that was previously published. Participants were directed to use the electronic HBB VR simulation and digitized HBB Provider’s Guide or the digitized HBB Provider’s Guide alone for a minimum of 20 minutes. HBB knowledge and skills assessments were then conducted, which were immediately followed by a standard, in-person HBB training course that was led by study staff and used standard HBB evaluation tools and the Neonatalie Live manikin (Laerdal Medical).

**Results:**

A total of 179 nurses and midwives participated (VR group: n=91; control group: n=88). The overall performance scores on the knowledge check (*P*=.29), bag and mask ventilation skills check (*P*=.34), and Objective Structured Clinical Examination A checklist (*P*=.43) were similar between groups, with low overall pass rates (6/178, 3.4% of participants). During the Objective Structured Clinical Examination A test, participants in the VR group performed better on the critical step of positioning the head and clearing the airway (VR group: 77/90, 86%; control group: 57/88, 65%; *P*=.002). The median percentage of ventilations that were performed via head tilt, as recorded by the Neonatalie Live manikin, was also numerically higher in the VR group (75%, IQR 9%-98%) than in the control group (62%, IQR 13%-97%), though not statistically significantly different (*P*=.35). Participants in the control group performed better on the *identifying a helper and reviewing the emergency plan* step (VR group: 7/90, 8%; control group: 16/88, 18%; *P*=.045) and the *washing hands* step (VR group: 20/90, 22%; control group: 32/88, 36%; *P*=.048).

**Conclusions:**

The use of digital interventions, such as mobile VR simulations, may be a viable approach to precourse preparation in neonatal resuscitation training for health care professionals in low- and middle-income countries.

## Introduction

Intrapartum asphyxia—the failure to breathe at birth—is a common medical emergency that occurs in the newborn period, and newborns with this condition require neonatal resuscitation to survive. The outcome of neonatal resuscitation depends on the availability of equipment and appropriately trained personnel [1]. The periodic in-service training of health care professionals, such as physicians, nurses, and midwives, on newborn resuscitation has significantly decreased neonatal mortality, which accounts for 46% of under-5 mortality globally [2-7]. Neonatal resuscitation training has largely depended on in-person, daylong workshops and manikin-based simulation exercises that are time-consuming and cost-intensive, resulting in widely spaced intervals for refresher training [4].

Newer models of neonatal resuscitation training involving the use of emerging technologies have been described [8,9]. Virtual reality (VR) is a new technology that has been described as “the learning aid of the 21st century,” as the feasibility and applicability of VR have been demonstrated in nearly all aspects of training and education [10,11]. The use of VR has provided engaging, individualized, and incentivized practice opportunities within immersive experiences [12], particularly under conditions like the COVID-19 pandemic, during which social distancing is encouraged.

Mobile VR simulations can be used to teach abstract ideas, illustrate real-world phenomena, and motivate students. [13]. The unpredictability of how and when neonatal resuscitation will be encountered in the clinical setting requires constant preparedness and confidence building, which can be gained via repeated practice (individually, in pairs, or as a small group). Due to the high level of user involvement in VR simulations, users may be exposed to materials more than once, lengthening the time spent actively learning and enhancing skill acquisition and retention. [9,14]. By connecting offline identities, game scenarios, and actual interactions with and within a virtual system, game-based learning enables learners to display abilities and alter behaviors that are related to clinical practice [15-19]. Evidence suggests that simulation games increase posttraining self-efficacy by 20%, declarative knowledge by 11%, procedural knowledge by 14%, and retention by 9% [20].

Although most software simulations require a PC with sufficient graphics capabilities or an advanced VR headset, mobile VR simulations can be delivered on mobile phones via a low-cost VR headset [9,21,22]. Health care professionals in low- and middle-income countries (LMICs) now have practically universal access to mobile phones, which encourages the accessibility, scalability, flexibility, and effectiveness of e-learning while lowering marginal costs. For instance, over the past 20 years, mobile subscriptions and broadband penetration have dramatically expanded in Nigeria [23]. According to the Nigerian Communications Commission, in 2020, there were 300 million connected mobile lines and 204 million active subscribers to Global System for Mobile Communications networks in Nigeria, resulting in a teledensity (the number of telephone connections) of 107 for every 100 individuals living in Nigeria [23].

This study involved a secondary analysis of data that were collected during the electronic Helping Babies Breathe (eHBB)/mobile Helping Babies Survive (mHBS) trial [24]. We explored the feasibility and educational efficacy of using mobile VR training and the digitized Helping Babies Breathe (HBB) Provider’s Guide, compared to using the digitized HBB Provider’s Guide alone, as an approach to precourse preparation for health care professionals attending in-person HBB courses in a low-resource setting. We hypothesized that, as measured via precourse assessments using validated HBB evaluation instruments, health care professionals who used mobile VR simulations and the digitized HBB Provider’s Guide before the course would be better prepared for in-person HBB training compared to those who used only the digitized guide without exposure to VR scenarios.

## Methods

### Study Design

This was a substudy of the prospective randomized controlled trial of an educational intervention, which was described fully elsewhere [24].

### Study Population

Health care professionals (nurses or nurse-midwives) who worked in labor, delivery, and newborn care units at 20 secondary and tertiary health care facilities in Lagos, Nigeria, and Busia, Western Kenya, and had not received training in HBB within the previous 1 year were recruited to participate in the eHBB study [24].

### Randomization

**Figure 1:**
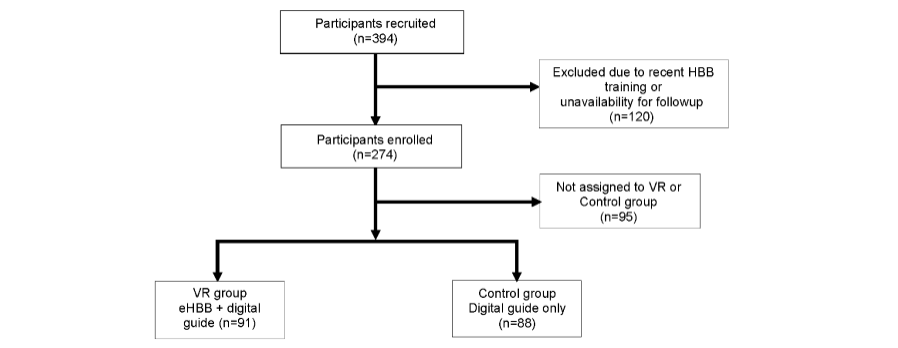
CONSORT (Consolidated Standards of Reporting Trials) flow chart. eHBB: electronic Helping Babies Breathe; HBB: Helping Babies Breathe; VR: virtual reality.

Participants were consented and were randomized to receive the eHBB and digitized HBB Provider’s Guide (VR group) intervention or the digitized HBB Provider’s Guide–only intervention (control group) before a standard in-person HBB course (Figure 1).

### Intervention

The eHBB VR simulations consisted of 3 brief, 3- to 5-minute, interactive 3D simulation scenarios that represented a newborn requiring routine care, some resuscitation, or prolonged resuscitation via positive pressure ventilation (Multimedia Appendix 1). The simulations were accessed using a low-cost VR headset and the eHBB virtual simulation app installed on participants’ study phones. The digitized HBB Provider’s Guide consisted of a digital version of the standard HBB, Second Edition, Provider’s Guide on the mHBS powered by District Health Information Software 2 (DHIS2; mHBS/DHIS2) app that was installed on participants’ phones. The VR group had access to the VR intervention and the digitized HBB Provider’s Guide. The mobile simulation scenarios were brief, and each could be completed within 3 to 5 minutes. A minimum of 20 minutes was given for the participants to familiarize themselves with their study group materials. Although they were encouraged to familiarize themselves with the eHBB VR simulation, the VR group, at enrollment, was allowed to access all of their study group materials (the eHBB VR simulation and digitized HBB Provider’s Guide) without restrictions. The control group only used the digitized HBB Provider’s Guide.

Precourse knowledge and skills assessments were then conducted by trained study staff using the following standardized tools: the HBB, Second Edition, knowledge check multiple-choice questionnaire; the bag and mask ventilation (BMV) skills check (a passing score was defined as correctly performing all 14 steps); and the Objective Structured Clinical Examination (OSCE) A checklist (a passing score was defined as correctly performing at least 9 of the 12 steps, including the required actions [items 4, 5, and 9]). The outcomes, which were measured by using the BMV skills check and the OSCE A checklist, were the health care professionals’ neonatal resuscitation skills before the standard in-person HBB, Second Edition, training course. The Neonatalie Advanced manikin (Laerdal Medical) was used for the standardized collection of data on BMV skills. Participants then received standard in-person HBB, Second Edition, training. Postcourse and follow-up evaluations for up to 6 months were described previously [24].

### Ethics Approval

This study was approved by the University of Lagos Health Research Ethics Committee (reference number: CMUL/HREC/09/18/445); Moi University Institutional Research Ethics Committee, Indiana University Institutional Review Board (reference number: 1807371465); and University of Washington Institutional Review Board (reference number: STUDY00005297).

### Data Analysis

Data were collected in real time by trained research assistants using the mHBS/DHIS2 mobile app [25] on dedicated Android mobile devices, and paper data forms were used as backups. A post hoc data analysis was performed for this study. Data were analyzed by using SAS version 9.4 (SAS Institute) and R version 4.0.0 (R Foundation for Statistical Computing) software. BMV performance data on the Neonatalie Live manikin were transmitted from the manikin to an iPad (Apple Inc) via Bluetooth and stored in a secure cloud database. Continuous variables were presented as means with SDs or medians with IQRs, and categorical variables were presented as numbers with percentages. The Kruskal-Wallis test or Wilcoxon rank-sum test was used to compare scores from the knowledge check, BMV skills test, and OSCE A test between groups. The Fisher exact test was used to compare overall test pass rates and pass rates for individual test items of interest. The sample size was not chosen specifically for this substudy but was initially determined to meet the goals of the primary study—to detect a 20% difference in pass rates on the OSCE B test between groups at the 6-month follow-up evaluation time point with 80% power [24]. Differences were considered significant when the 2-sided *P* values were <.05.

## Results

### Demographic Characteristics

Data from 179 health care professionals who were assigned to the VR and control groups were reviewed. Table 1 shows the demographic characteristics of the study participants, which were similar between the intervention (VR and digital guide) and control (digital guide only) groups. Most of the participants were female (162/179, 90.5%). Of the 179 participants, only 29 (16.2%) had ever undergone training on HBB, and all previously trained health care professionals were trained with the HBB, First Edition, curriculum. Nearly all of the participants (163/179, 91.1%) owned a smartphone.

The median scores (out of 18) on the precourse knowledge check were similar for both groups (VR group: 15, IQR 14-16; control group: 16, IQR 15-17; *P*=.29).

**Table 1:** Demographic characteristics of the study participants.

Characteristic		Group	
		VR^a^ (n=91)	Control (n=88)
Age (years), mean (SD)		37 (9)	37 (9)
**Sex, n (%)**			
	Male	9 (10)	8 (9)
	Female	82 (90)	80 (91)
**Profession, n (%)**			
	Nurse	43 (47)	44 (50)
	Nurse-midwife	48 (53)	44 (50)
**Country, n (%)**			
	Kenya	44 (48)	42 (48)
	Nigeria	47 (52)	46 (52)
**Ward^b^, n (%)**			
	Labor or delivery ward	62 (71)	63 (73)
	Postnatal ward	15 (17)	18 (21)
	NBU^c^ or NICU^d^	6 (7)	4 (5)
	Operating theater	4 (5)	1 (1)
**Years of postqualification experience^b^, n (%)**			
	<5	22 (24)	26 (30)
	5-10	34 (38)	25 (28)
	11-15	11 (12)	13 (15)
	16-20	11 (12)	8 (9)
	>20	12 (13)	16 (18)
Prior HBB^e^ training, n (%)		16 (18)	13 (15)
Owns a smartphone, n (%)		81 (89)	82 (93)

^a^VR: virtual reality.

^b^Participants with missing values were excluded from the corresponding summary (ward: n=12; years of experience: n=2).

^c^NBU: newborn unit.

^d^NICU: neonatal intensive care unit.

^e^HBB: Helping Babies Breathe.

### BMV Skills

The overall performance on the precourse BMV skills check was also similar between groups, with a median score (out of 14) of 4.93 (IQR 3-6) in the VR group and 5.32 (IQR 4-7) in the control group (*P*=34). Among all participants, 1 participant in the VR group achieved a passing score of 14. There were no statistically significant differences in the pass rates for individual test items between the VR and control groups, as shown in Table 2.

**Table 2:** Precourse bag and mask ventilation performance.

Steps	Group^a^, n (%)		*P* value
	Virtual reality (n=83)	Control (n=79)	
Place baby on ventilation area	49 (59)	45 (57)	.87
Stand at the baby’s head	38 (46)	45 (57)	.16
Check that the mask size is correct	32 (39)	39 (49)	.21
Position the head slightly extended	41 (49)	40 (51)	>.99
Apply the mask to the face	57 (69)	58 (73)	.60
Make a tight seal	25 (30)	20 (25)	.60
Squeeze the bag	25 (30)	16 (20)	.21
Ventilate	22 (27)	20 (25)	>.99
Ventilate at 40 breaths per minute	13 (16)	10 (13)	.66
Reapply mask	39 (47)	44 (56)	.28
Reposition head	36 (43)	40 (51)	.43
Clear mouth and nose of secretions	13 (16)	11 (14)	.83
Open the mouth	2 (2)	1 (1)	>.99
Squeeze the bag harder	17 (20)	19 (24)	.71

^a^In total, 83 of the 91 participants in the virtual reality group and 79 of the 88 participants in the control group had bag and mask ventilation skills check results available.

### OSCE A Performance

The median scores (out of 12) on the precourse OSCE A checklist were 5.91 (IQR 4-8) in the VR group and 5.83 (IQR 4-7) in the control group (*P*=.43). Only 4% (4/90) of the participants in the VR group and 2% (2/88) of the participants in the control group achieved passing scores. Participants’ performance on individual test items is shown in Table 3. The two steps that the control group more frequently performed were the *identifying a helper and reviewing the emergency plan* step (VR group: 7/90, 8%; control group: 16/88, 18%; *P*=.045) and the *washing hands* step (VR group: 20/90, 22%; control group: 32/88, 36%; *P*=.048). Participants in the VR group performed better on the critical step of positioning the head and clearing the airway (VR group: 77/90, 86%; control group: 57/88, 65%; *P*=.002). In addition, the VR group tended to perform better on the *removing the wet cloth* step than the control group (VR group: 34/90, 38%; control group: 22/88, 25%; *P*=.08).

The precourse data from the Neonatalie Live manikin showed that participants in both groups spent a median time of 160 seconds performing BMV, with a median ventilation rate of 29 (VR group: 29.4; control group: 29.3) breaths per minute. The median percentages of ventilations with low pressures (VR group: 4%, IQR 0%-12%; control group: 4%, IQR 0%-17%; *P*=.27), ventilations with high pressures (VR group: 0%, IQR 0%-9%; control group: 0%, IQR 0%-2%; *P*=.20), and valid ventilations (VR group: 19%, IQR 2%-59%; control group: 19%, IQR 0%-57%; *P*=.68) were similar between both groups. The median percentage of ventilations that were performed via head tilt was 75% (IQR 9%-98%) in the VR group and 62% (IQR 13%-97%) in the control group (*P*=.35).

**Table 3:** Precourse Objective Structured Clinical Examination A performance.

Steps	Group^a^, n (%)		*P* value
	Virtual reality (n=90)	Control (n=88)	
Identifies a helper and reviews the emergency plan	7 (8)	16 (18)	.045
Prepares the area for delivery	49 (54)	52 (59)	.55
Washes hands	20 (22)	32 (36)	.048
Prepares ventilation area	44 (49)	38 (43)	.46
Dries thoroughly	55 (61)	54 (61)	>.99
Removes wet cloth	34 (38)	22 (25)	.08
Recognizes baby is not crying	51 (57)	49 (56)	>.99
Positions head and clears airway	77 (86)	57 (65)	.002
Stimulates breathing by rubbing the back	23 (26)	24 (27)	.87
Recognizes baby is crying and breathing well	63 (70)	55 (62)	.34
Clamps or ties and cuts the cord	56 (62)	48 (55)	.36
Communicates with mother	46 (51)	40 (45)	.46

^a^In total, 90 of the 91 participants in the virtual reality group and all 88 participants in the control group had Objective Structured Clinical Examination A results available.

## Discussion

### Principal Findings

This study is the first to demonstrate the feasibility and educational efficacy of using mobile VR simulations and digital manuals as an approach to precourse preparation for health care professionals undergoing neonatal resuscitation training in a low-resource setting. The overall precourse performance on knowledge assessments was higher than the overall precourse performance on skills assessments. Although the precourse mobile VR simulation and the reading of a digital manual prior to training did not result in a substantial number of participants achieving a passing score on the HBB skills assessments that were administered before the session, the use of these materials may promote interest in learning. The group that only reviewed the digital manual demonstrated significantly better performance than the VR group on the OSCE A for the *identifying a helper and reviewing the emergency plan* step (*P*=.045) and *washing hands* step (*P*=.048) However, the performance of the critical *positioning the head and clearing the airway* step via head tilt to open the airway occurred more frequently in the VR group.

A growing body of evidence from well-designed studies supports the use of simulation to enhance clinical performance [1,8,20,26,27]. The use of VR simulation was associated with changes in stress physiology in a study by Chang et al [28]. Our precourse simulation introduces neonatal resuscitation to participants and may promote interest in learning. It may also save costs and shorten the in-person training time for participants who have already had an immersive experience with the HBB course [10,29].

Using mobile VR simulations can help individuals build skills and confidence for cognitive tasks; thus, such simulations complement formal in-person training that focuses on manikin-based psychomotor tasks, such as BMV [9,30]. The VR group performed better on specific cognitive tasks. Though not statistically significant (*P* values were >.05 for various tasks; Tables 2 and 3), this incremental benefit demonstrates the potential of VR training to support knowledge and performance skills. Those exposed to the VR simulation demonstrated better knowledge of when a child should be suctioned and other cognitive steps, such as removing the wet cloth and clamping and cutting the cord. They were more likely to recognize when the baby was not crying and when the baby was crying and breathing well. These steps are relevant to quickly initiating neonatal resuscitation and appropriately discontinuing resuscitation when the baby has responded, and they are taught at later points in the in-person HBB course curriculum.

Of note, the *identifying a helper and reviewing the emergency plan* step is featured early in the HBB digital guide, as it is a part of preparing for a delivery; thus, it is possible that this concept was more easily recalled by participants in the control group. Although this concept is also presented in the VR simulation, there is no specific action required by the learner, unlike in other more active elements of the simulation. Featuring a conversational helper in the VR simulation may help to emphasize this aspect of preparing for a delivery.

Communication with the mother is an important element of respectful care [31]. There is evidence that even if health care professionals are skilled in managing pregnancy and birth complications, women may refuse to seek care and discourage others from doing so when they have previously experienced disrespectful care [32]. Manikin-based simulations and VR simulations have been used to teach nontechnical skills and have been shown to improve communication attitudes [27,33,34]. We found that participants in the VR group communicated with the mother with greater frequency during the OSCE evaluation, thereby demonstrating that they recognized the importance of prospectively providing information and engaging with the mother via effective communication, which are essential elements of respectful care [32].

This study demonstrated the feasibility of using mobile VR simulations for precourse training on neonatal resuscitation in a low-resource setting. Computer-based training simulations, such as HeartCode Pediatric Acute Life Support and the Neonatal Resuscitation Program eSim programs, have been used to complement in-person courses in high-resource settings, with participants being assigned to perform the computer-based simulations up to 1 month before attending in-person courses [9,35,36]. Although simulation laboratories and equipment are lacking [37], the broad use of mobile devices in LMICs supports the need for innovations in the design and distribution of simulation education materials on mobile devices for self-directed learning [9,24]. Nearly all of the participants in our study (163/179, 91%) owned a smartphone. Mobile VR simulations are more accessible to learners than manikin-based simulations or computer-based simulations in low-resource settings, where mobile phones are widely used [23,24,38]. As the receptiveness to VR training is high, spending additional time in individualized precourse exposure may improve learners’ performance [39-41].

### Limitations to This Study

Some limitations may affect the interpretation of our results. First, although it would have been reasonable to conduct a baseline skills assessment prior to and after introducing the digital interventions to participants, this analysis was not preplanned, and due to the logistical constraints posed by multiple assessments, we performed precourse assessments in both the VR and control groups after their exposure to the digital interventions but prior to the in-person course. Second, although the VR scenarios were brief and could be completed within 3 to 5 minutes, the minimum time (20 minutes) allotted for digital intervention familiarization prior to the precourse knowledge and skills assessments may not have permitted participants to experience the full impact of the interventions. Increasing the pretraining VR exposure time may optimize learning and reduce in-person training time. Finally, mobile VR simulations may be more suitable for gaining cognitive skills and less suitable for gaining psychomotor skills or learning how to perform manual tasks. Nevertheless, this study provides a possible insight into the relative contribution of precourse exposure and the feasibility of undergoing a mobile VR simulation prior to neonatal resuscitation training in an LMIC setting. Future studies should explore the potential for cost savings or shorter in-person training times for participants who have already been exposed (ie, immersive exposure) to the HBB course content prior to the in-person training [10,29].

### Conclusions

The use of digital interventions, such as mobile VR, is feasible and may be a viable approach to precourse preparation in neonatal resuscitation training for health care professionals in LMICs. The role of mobile VR simulation should be further evaluated in the context of training health care professionals in low-resource settings, particularly when access to in-person training with manikin-based simulations is limited.

## Appendix

Multimedia Appendix 1 Helping Babies Breathe 2 virtual reality app demo (2020).

## Abbreviations

BMV: bag and mask ventilation
DHIS2: District Health Information Software 2
eHBB: electronic Helping Babies Breathe
HBB: Helping Babies Breathe
LMIC: low- and middle-income country
mHBS: mobile Helping Babies Survive
mHBS/DHIS2: mobile Helping Babies Survive powered by District Health Information Software 2
OSCE: Objective Structured Clinical Examination
VR: virtual reality

## Data Availability

Data are available on reasonable request. Deidentified data are available on request from RU (nestprog@uw.edu).

## References

[1] Garvey AA, Dempsey EM (2020). Simulation in neonatal resuscitation. Front Pediatr.

[2] Perin J, Mulick A, Yeung D, Villavicencio F, Lopez G, Strong KL, Prieto-Merino D, Cousens S, Black RE, Liu L (2022). Global, regional, and national causes of under-5 mortality in 2000-19: an updated systematic analysis with implications for the Sustainable Development Goals. Lancet Child Adolesc Health.

[3] Reisman J, Arlington L, Jensen L, Louis H, Suarez-Rebling D, Nelson BD (2016). Newborn resuscitation training in resource-limited settings: A systematic literature review. Pediatrics.

[4] Pammi M, Dempsey EM, Ryan CA, Barrington KJ (2016). Newborn resuscitation training programmes reduce early neonatal mortality. Neonatology.

[5] Matendo R, Engmann C, Ditekemena J, Gado J, Tshefu A, Kinoshita R, McClure EM, Moore J, Wallace D, Carlo WA, Wright LL, Bose C (2011). Reduced perinatal mortality following enhanced training of birth attendants in the Democratic Republic of Congo: a time-dependent effect. BMC Med.

[6] (2021). Neonatal mortality. UNICEF Data.

[7] Niermeyer S (2015). From the Neonatal Resuscitation Program to Helping Babies Breathe: Global impact of educational programs in neonatal resuscitation. Semin Fetal Neonatal Med.

[8] Bucher SL, Cardellichio P, Muinga N, Patterson JK, Thukral A, Deorari AK, Data S, Umoren R, Purkayastha S (2020). Digital health innovations, tools, and resources to support Helping Babies Survive programs. Pediatrics.

[9] Ghoman SK, Patel SD, Cutumisu M, von Hauff P, Jeffery T, Brown MRG, Schmölzer GM (2020). Serious games, a game changer in teaching neonatal resuscitation? A review. Arch Dis Child Fetal Neonatal Ed.

[10] Rogers S (2019). Virtual reality: The learning aid of the 21st century. Forbes.

[11] Radianti J, Majchrzak TA, Fromm J, Wohlgenannt I (2020). A systematic review of immersive virtual reality applications for higher education: Design elements, lessons learned, and research agenda. Comput Educ.

[12] Sheik-Ali S, Edgcombe H, Paton C (2019). Next-generation virtual and augmented reality in surgical education: A narrative review. Surg Technol Int.

[13] Vishwanath A, Kam M, Kumar N (2017). Examining low-cost virtual reality for learning in low-resource environments.

[14] Murphy AA, Halamek LP (2005). Educational perspectives: Simulation-based training in neonatal resuscitation. Neoreviews.

[15] Waggoner Z (2009). My Avatar, My Self: Identity in Video Role-Playing Games.

[16] Parsons TD, Rizzo AA (2008). Affective outcomes of virtual reality exposure therapy for anxiety and specific phobias: a meta-analysis. J Behav Ther Exp Psychiatry.

[17] Seymour NE (2008). VR to OR: a review of the evidence that virtual reality simulation improves operating room performance. World J Surg.

[18] Robb A, White C, Cordar A, Wendling A, Lampotang S, Lok B (2015). A comparison of speaking up behavior during conflict with real and virtual humans. Comput Human Behav.

[19] Shorey S, Ng ED (2021). The use of virtual reality simulation among nursing students and registered nurses: A systematic review. Nurse Educ Today.

[20] Sitzmann T (2011). A meta-analytic examination of the instructional effectiveness of computer-based simulation games. Pers Psychol.

[21] Nagunwa TP, Lwoga ET (2012). Developing an eLearning strategy to implement medical competency based curricula: Experiences from Muhimbili University of Health and Allied Sciences. Int J Educ Dev Using Inf Commun Technol.

[22] Umoren RA, Bucher S, Purkayastha S, Ezeaka C, Esamai F, Mairami A, Asangansi I, Bresnahan B, Paton C (2020). eHBB/mHBS-DHIS2: mobile virtual reality provider training in Helping Babies Breathe. Pediatrics.

[23] Subscriber data. Nigerian Communications Commission.

[24] Umoren R, Bucher S, Hippe DS, Ezenwa BN, Fajolu IB, Okwako FM, Feltner J, Nafula M, Musale A, Olawuyi OA, Adeboboye CO, Asangansi I, Paton C, Purkayastha S, Ezeaka CV, Esamai F (2021). eHBB: a randomised controlled trial of virtual reality or video for neonatal resuscitation refresher training in healthcare workers in resource-scarce settings. BMJ Open.

[25] Bucher S, Meyers E, Kshatriya BSA, Avanigadda PC, Purkayastha S (2019). Development of an innovative mobile phone-based newborn care training application. Innovations in Bio-Inspired Computing and Applications.

[26] Umoren R, Ezeaka VC, Fajolu IB, Ezenwa BN, Akintan P, Chukwu E, Spiekerman C (2020). Perspectives on simulation-based training from paediatric healthcare providers in Nigeria: a national survey. BMJ Open.

[27] Bender J, Kennally K, Shields R, Overly F (2014). Does simulation booster impact retention of resuscitation procedural skills and teamwork?. J Perinatol.

[28] Chang TP, Hollinger T, Dolby T, Sherman JM (2021). Development and considerations for virtual reality simulations for resuscitation training and stress inoculation. Simul Healthc.

[29] Krokos E, Plaisant C, Varshney A (2018). Virtual memory palaces: immersion aids recall. Virtual Real.

[30] Horwood C, Haskins L, Luthuli S, McKerrow N (2019). Communication between mothers and health workers is important for quality of newborn care: a qualitative study in neonatal units in district hospitals in South Africa. BMC Pediatr.

[31] Nassar AK, Al-Manaseer F, Knowlton LM, Tuma F (2021). Virtual reality (VR) as a simulation modality for technical skills acquisition. Ann Med Surg (Lond).

[32] Shakibazadeh E, Namadian M, Bohren MA, Vogel JP, Rashidian A, Pileggi VN, Madeira S, Leathersich S, Tunçalp Ӧ, Oladapo OT, Souza JP, Gülmezoglu AM (2018). Respectful care during childbirth in health facilities globally: a qualitative evidence synthesis. BJOG.

[33] Sweigart LI, Umoren RA, Scott PJ, Carlton KH, Jones JA, Truman B, Gossett EJ (2016). Virtual TeamSTEPPS(®) simulations produce teamwork attitude changes among health professions students. J Nurs Educ.

[34] Bracq MS, Michinov E, Jannin P (2019). Virtual reality simulation in nontechnical skills training for healthcare professionals: A systematic review. Simul Healthc.

[35] Sawyer T, Umoren RA, Gray MM (2016). Neonatal resuscitation: advances in training and practice. Adv Med Educ Pract.

[36] Gray MM, Umoren RA, Josephsen J, Chitkara R, Strand M, Ramachandran S, Weiner G, Zaichkin J, Sawyer T, Pantone G, Billimoria Z, Cabrera AK, Motz P, Sie L, Weiner Y, Kan P, Stavroudis T, Ades A, Lee HC (2020). Gaps in neonatal provider performance on standardized simulations: A report from the NRP eSim study. Pediatrics.

[37] Turkot O, Banks MC, Lee SW, Dodson A, Duarte S, Kaino M, Nelson-Williams H, Toy S, Sampson J (2019). A review of anesthesia simulation in low-income countries. Curr Anesthesiol Rep.

[38] Frehywot S, Vovides Y, Talib Z, Mikhail N, Ross H, Wohltjen H, Bedada S, Korhumel K, Koumare AK, Scott J (2013). E-learning in medical education in resource constrained low- and middle-income countries. Hum Resour Health.

[39] Lange AK, Koch J, Beck A, Neugebauer T, Watzema F, Wrona KJ, Dockweiler C (2020). Learning with virtual reality in nursing education: Qualitative interview study among nursing students using the Unified Theory of Acceptance and Use of Technology model. JMIR Nurs.

[40] Moore N, Yoo S, Poronnik P, Brown M, Ahmadpour N (2020). Exploring user needs in the development of a virtual reality-based advanced life support training platform: Exploratory usability study. JMIR Serious Games.

[41] Kyaw BM, Saxena N, Posadzki P, Vseteckova J, Nikolaou CK, George PP, Divakar U, Masiello I, Kononowicz AA, Zary N, Car LT (2019). Virtual reality for health professions education: Systematic review and meta-analysis by the Digital Health Education Collaboration. J Med Internet Res.

