# Supplementary material for: Using Mobile Virtual Reality Simulation to Prepare for In-Person Helping Babies Breathe Training: Secondary Analysis of a Randomized Controlled Trial (the eHBB/mHBS Trial)
Source: JMIR Med Educ. 2022 Sep 12;8(3):e37297. doi: 10.2196/37297 (PMC9513689; doi:10.2196/37297)
Supplement: Multimedia Appendix 1 [file mededu_v8i3e37297_app1.docx]

[
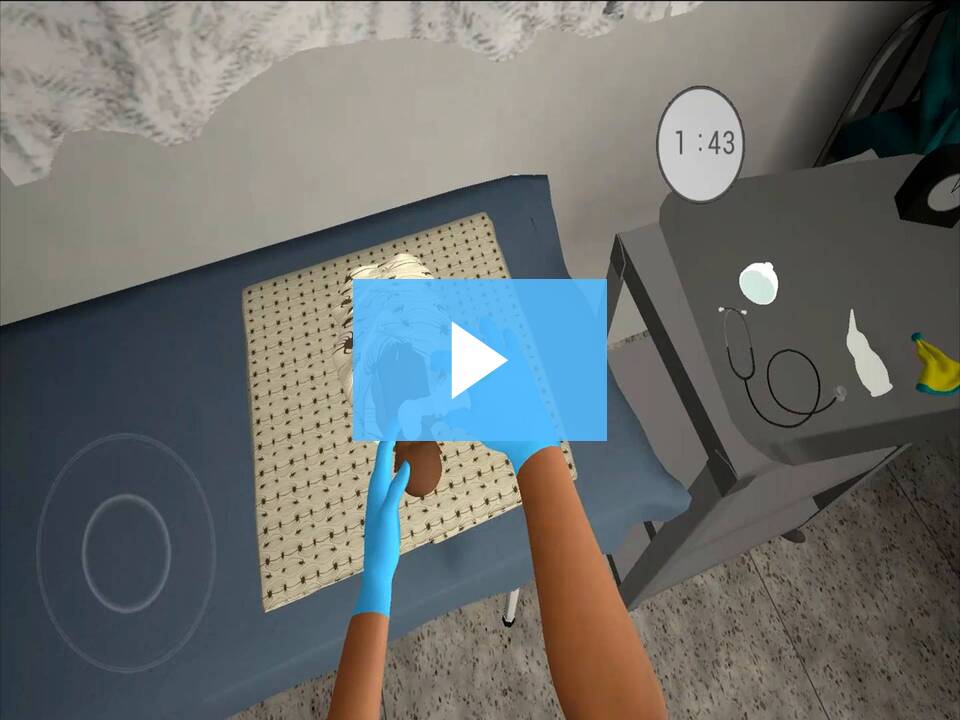
](https://nestprogram.wistia.com/medias/5mk2yz2c8o?wvideo=5mk2yz2c8o)

<https://nestprogram.wistia.com/medias/5mk2yz2c8o>
